# Oxidative stress–induced autonomous activation of the calcium/calmodulin-dependent kinase II involves disulfide formation in the regulatory domain

**DOI:** 10.1016/j.jbc.2022.102579

**Published:** 2022-10-08

**Authors:** Nathália Rocco-Machado, Lo Lai, Geumsoo Kim, Yi He, Elizabeth D. Luczak, Mark E. Anderson, Rodney L. Levine

**Affiliations:** 1 Laboratory of Biochemistry, National Heart, Lung and Blood Institute, Bethesda, Maryland, USA; 2 Fermentation Facility, National Heart, Lung, and Blood Institute, National Institutes of Health, Bethesda, Maryland, USA; 3 Division of Cardiology, Department of Medicine, Johns Hopkins University, Baltimore, Maryland, USA; 4 Department of Physiology and Program in Cellular and Molecular Medicine, The Johns Hopkins University School of Medicine, Baltimore, Maryland, USA; 5 Department of Genetic Medicine, The Johns Hopkins University School of Medicine, Baltimore, Maryland, USA

**Keywords:** CaMKII, oxidative stress, disulfide, redox regulation, cysteine, methionine, CaMKII, calcium/calmodulin-dependent protein kinase II, IAA, iodoacetamide, TCA, trichloroacetic acid

## Abstract

Calcium/calmodulin-dependent protein kinase II δ (CaMKIIδ) has a pivotal role in cardiac signaling. Constitutive and deleterious CaMKII “autonomous” activation is induced by oxidative stress, and the previously reported mechanism involves oxidation of methionine residues in the regulatory domain. Here, we demonstrate that covalent oxidation leads to a disulfide bond with Cys273 in the regulatory domain causing autonomous activity. Autonomous activation was induced by treating CaMKII with diamide or histamine chloramine, two thiol-oxidizing agents. Autonomy was reversed when the protein was incubated with DTT or thioredoxin to reduce disulfide bonds. Tryptic mapping of the activated CaMKII revealed formation of a disulfide between Cys273 and Cys290 in the regulatory domain. We determined the apparent pKa of those Cys and found that Cys273 had a low pKa while that of Cys290 was elevated. The low pKa of Cys273 facilitates oxidation of its thiol to the sulfenic acid at physiological pH. The reactive sulfenic acid then attacks the thiol of Cys290 to form the disulfide. The previously reported CaMKII mutant in which methionine residues 281 and 282 were mutated to valine (MMVV) protects mice and flies from cardiac decompensation induced by oxidative stress. Our initial hypothesis was that the MMVV mutant underwent a conformational change that prevented disulfide formation and autonomous activation. However, we found that the thiol-oxidizing agents induced autonomy in the MMVV mutant and that the mutant undergoes rapid degradation by the cell, potentially preventing accumulation of the injurious autonomous form. Together, our results highlight additional mechanistic details of CaMKII autonomous activation.

The Ca^2+^/calmodulin dependent protein kinase II (CaMKII) is a multifunctional serine/threonine kinase comprised of 12 monomers that form a dodecameric holoenzyme (1, 2, 3). CaMKIIδ, the dominant isoform in the heart, has a central role in intracellular Ca^2+^ regulation and modulation of excitation-contraction coupling (4, 5), gene transcription (6, 7), mitochondrial reprogramming (8, 9, 10), inflammasome activation (11, 12), and apoptosis (13, 14). Each CaMKII monomer consists of three domains: (I) a catalytic domain in the amino terminal region containing the ATP-binding site; (II) a regulatory domain that mediates the function of the catalytic domain; and (III) a carboxy-terminal association domain that drives multimeric assembly (1, 2, 3). The regulatory domain autoinhibits the kinase activity under resting conditions. Binding of calcified calmodulin (Ca^2+^/calmodulin) induces a conformational change that relieves the inhibition. Inhibition is restored as Ca^2+^ undergoes reuptake and calmodulin dissociates (1, 2, 3). However, a sustained activation of CaMKIIδ induces an autophosphorylation at Thr287 that generates Ca^2+^/calmodulin-independent enzyme activity (autonomy) by preventing reassociation of the kinase domain (1, 15). Sustained, autonomous kinase activity promotes arrythmias, cardiomegaly, cardiomyocyte apoptosis, cardiac failure, and sudden death (16). Inhibition of CaMKII is a highly successful preclinical intervention for treating or preventing adverse outcomes in many cardiovascular disease models (17). CaMKII is thus an important therapeutic target and CaMKII inhibition may reduce or prevent heart failure induced by chronic oxidative stress (18, 19, 20).

Oxidation of CaMKII can cause autonomy (21) and oxidized CaMKII is implicated in cardiovascular disease (17), pulmonary disease (22), and cancer (23). It was reported Met281/282 to be the target of oxidation in CaMKII (22, 24) and knockin mice (MMVV) lacking Met281/282 are resistant to heart failure, arrhythmias (25, 26, 27), and asthma (28). The proposal that Met281/282 were oxidized was supported, in part, by studies made with an antiserum raised against a peptide exposed to micromolar H_2_O_2_ (residues 273–289, CQRSTVASMMHRQETVD, that span much of the regulatory region of CaMKII) (24). Numerous publications from different laboratories have used the antiserum to detect oxidatively modified CaMKII in both *in vitro* and *in vivo* experiments (18, 25, 28, 29, 30, 31). Those papers establish that elevated oxidized CaMKII, as detected by the antiserum, is induced by oxidative stress and correlates with cardiac failure and pathology. However, the CaMKII peptide used to raise the antiserum also contained a cysteine at its amino terminus (Fig. 1*A*). Published rate constants for the reaction of methionine or cysteine with 100 μM H_2_O_2_ for 1 h predict that less than 0.3% of the methionine could be oxidized to the sulfoxide in the absence of a catalytic process while >80% would be oxidized to form a disulfide-linked dimeric peptide (32, 33) (Fig. 1, *B* and *C*). Here, we show that the antiserum against oxidized CaMKII recognizes disulfide formation and not methionine oxidation. We also show that the disulfide links Cys273 and Cys290 in the regulatory domain, thus inducing autonomous activity of CaMKII.

**Figure 1:**
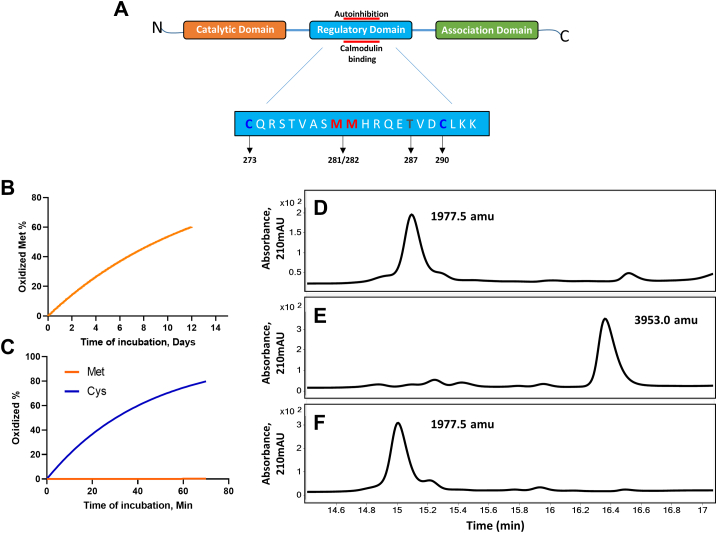
**CaMKII peptide (CQRSTVASMMHRQETVD) oxidized by H**_**2**_**O**_**2**_**forms a disulfide-linked dimeric peptide.***A*, general structure of a CaMKII monomer. The amino acid sequence of the regulatory domain is highlighted. *B*, calculated rate of Met or (*C*) Met and Cys oxidized by H_2_O_2_. *D*, chromatogram and measured mass of the native CaMKII peptide using HPLC-MS/MS (representative image of n = 2 replicates). *E*, CaMKII peptide treated with H_2_O_2_ (representative image of n = 2 replicates). *F*, CaMKII peptide treated with H_2_O_2_ and DTT (representative image of n = 2 replicates). CaMKII, calcium/calmodulin-dependent protein kinase II; mAU, milliabsorbance units.

## Results

### Antiserum against oxidized CaMKII recognizes disulfide formation and not methionine oxidation

In order to determine whether the immunogen used to raise the anti-oxidized CaMKII antiserum was actually a disulfide linked peptide, we synthesized the same peptide used in the initial description of CaMKII oxidation (24), incubated it with 100 μM H_2_O_2_ for 1 h, and analyzed the product by HPLC-MS/MS. The peptide native mass is 1977.5 Da (Fig. 1*D*) and after incubation with H_2_O_2_, we found only the disulfide-linked dimeric peptide without methionine oxidation (Fig. 1*E*). The native monomer was regenerated by incubation with DTT (Fig. 1*F*).

We then evaluated the specificity of the anti-oxidized CaMKII serum (Fig. S1). We prepared the reduced monomeric peptide (Fig. S1*A*), the monomeric (Fig. S1*B*) and disulfide-linked peptides with both methionine residues oxidized to the sulfoxide (Fig. S1*C*), and the disulfide-linked peptide without methionine sulfoxide (Fig. S1*D*). Antiserum linked to magnetic beads was incubated with each peptide and their binding determined by HPLC-mass spectrometry. Only the disulfide-linked peptide without methionine sulfoxide bound to the antiserum (Fig. S1*D*). We also prepared the CaMKII peptide in disulfide-linkage with GSH and with three randomly selected cysteine peptides. The antiserum did not bind to the GSH-modified peptide nor to two of the three randomly selected peptides (Fig. S1, *E* and *F*). It did bind to the third peptide that was about the same length as the CaMKII peptide (Fig. S1*G*). We conclude that the antiserum raised against the H_2_O_2_-exposed peptide detects a disulfide-containing CaMKII and not Met281/Met282 sulfoxide.

### Two cysteines in the CaMKII regulatory domain are solvent exposed: Cys273 and Cys290

**Figure 2:**
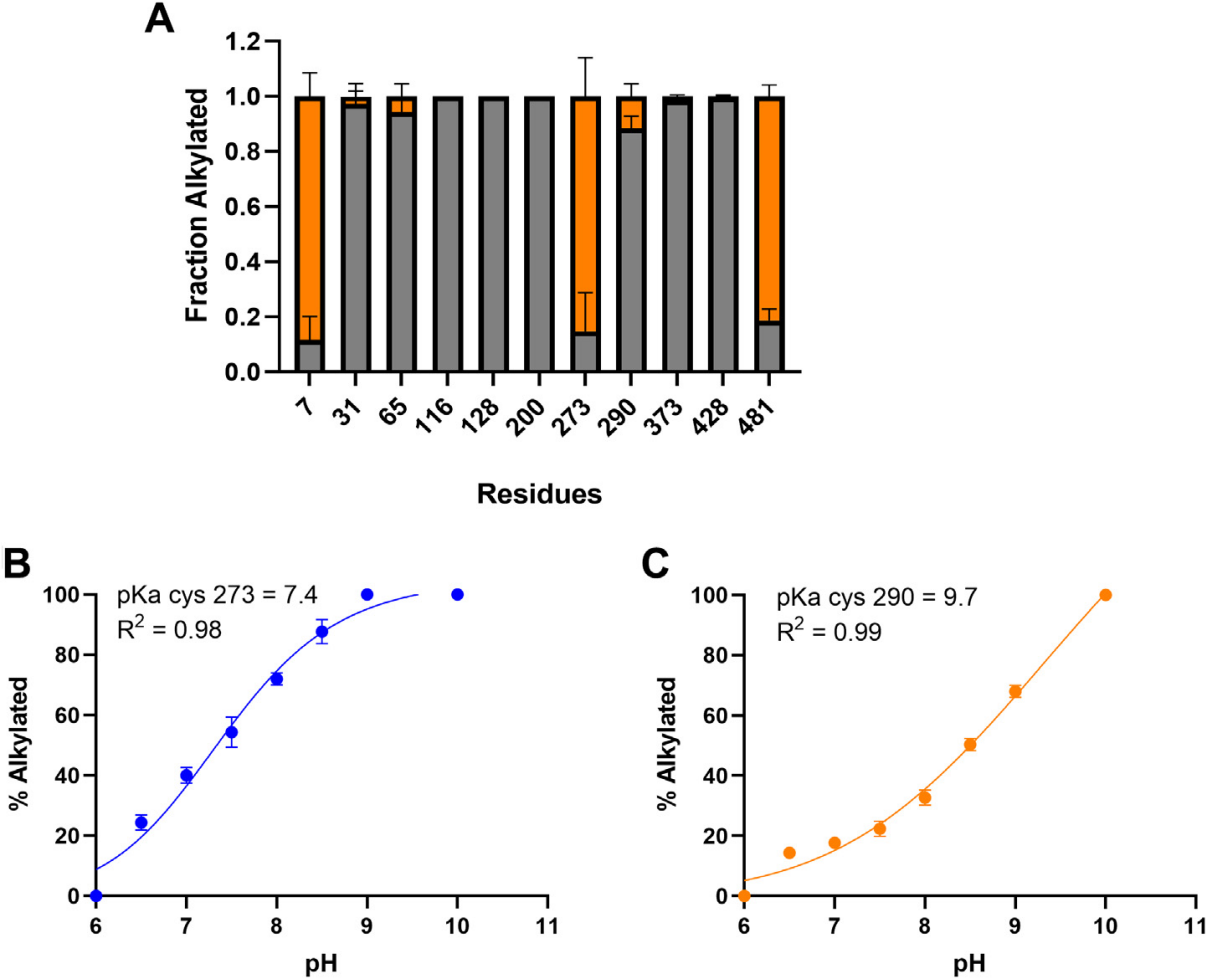
**CaMKII has four exposed cysteines, two of which are in the regulatory domain: Cys273 and Cys290.***A*, purified CaMKII was incubated with iodoacetamide to label exposed cysteine residues (*orange color*). After that, CaMKII was denatured and reduced by DTT. Acrylamide was added to label the free cysteines (*gray color*). The enzyme was incubated with trypsin and the mapping was done on HPLC-MS. The experiment was replicated three times. *B*, the Cys273 mutant or (*C*) Cys290 mutant were incubated with iodoacetamide at different pHs as indicated in the figure. The percent of derivatized protein molecules was determined by mass spectrometry. The experiment was replicated three times. The curves were fit by Prism to an allosteric sigmoidal. CaMKII, calcium/calmodulin-dependent protein kinase II.

**Figure 3:**
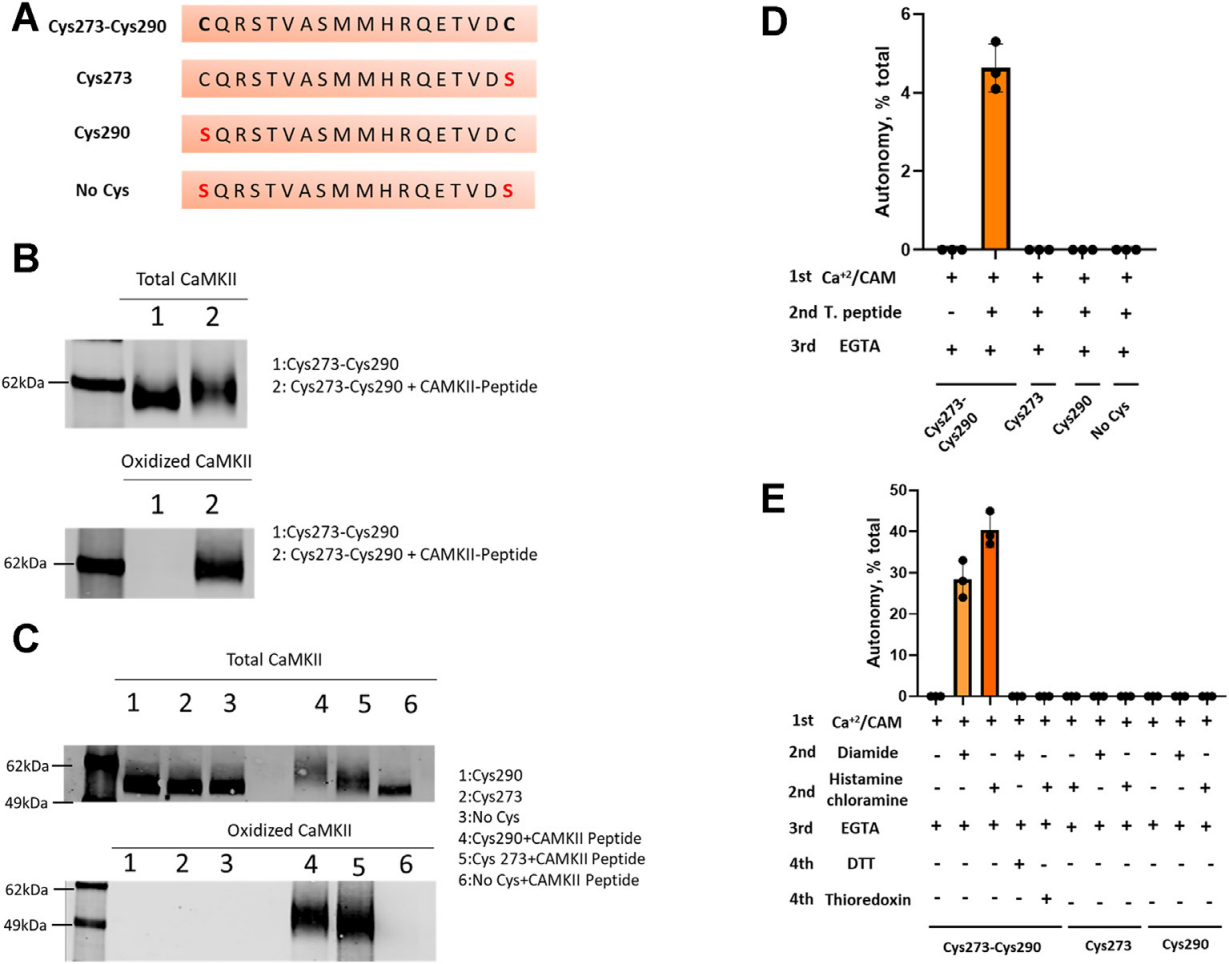
**Disulfide formation in the CaMKII regulatory domain induces autonomy *in vitro* only if both Cys273 and Cys290 are present in the protein.***A*, CaMKII mutants' sequences and (*B*) purified CaMKII Cys273-Cys290 mutant was incubated or not with the thiopyridylated-CaMKII peptide (CaMKII-peptide) and probed with anti-CaMKII antibody (*upper panel*) or anti-oxidized CaMKII antibody (*lower panel*). Representative image of n = 3 replicates. *C*, purified CaMKII Cys273, Cys290, and No Cys mutants were incubated or not with the thiopyridylated-CaMKII peptide and probed with anti-CaMKII antibody (*upper panel*) or anti-oxidized CaMKII antibody (*lower panel*). Representative image of n = 3 replicates. *D*, purified CaMKII Cys273-Cys290, Cys273, Cys290, and No Cys mutants derivatized or not with the thiopyridylated-CaMKII peptide autonomous activity (after EGTA addition). *E*, purified CaMKII Cys273-Cys290, Cys273, and Cys290 mutants incubated or not with diamide or histamine chloramine in the presence or not of the reducing agents DTT or thioredoxin autonomous activity (after EGTA addition). n = 3 replicates. % of total activity (No EGTA addition). CaMKII, calcium/calmodulin-dependent protein kinase II.

There are 11 cysteines in CaMKII, and the available three-dimensional structures suggest that four of these are solvent exposed and thus particularly susceptible to oxidation. Cys7, in the amino terminus, is in a natively unfolded domain ((34) and PDB 6AYW). Cys481, near the carboxy terminus, is also located in a natively unfolded region and that region is typically deleted in order to obtain crystals for structure determination. Two other cysteine residues, Cys273 and Cys290, are solvent exposed in the available structures ((34), 6AYW). The solvent exposure of these four cysteines, and only those four, was established by the ability of iodoacetamide (IAA) to alkylate them (Fig. 2*A*). Unless otherwise stated, all studies reported below were with CaMKIIδ in which Cys7 and Cys481 were mutated to Ser to avoid disulfide formation by them. The catalytic activity of the C7S/C481S was the same as that of WT enzyme (Fig. S2). For clarity, we named mutants by the regulatory domain cysteine residues that they contain (Fig. 3*A*). For example, C7S/C290S/C481S is named Cys273.

We determined the apparent pK_a_ of Cys273 and Cys290 because Cys with lower pK_a_ have heightened nucleophilicity and are more readily oxidized (32) (Fig. 2, *B* and *C*). Specific site mutations were made to generate CaMKII mutants containing only Cys273 or Cys290. No difference in the CaMKII Ca^2+^/Calmodulin-dependent activity was observed between the WT and the CaMKII mutants (Fig. S2). The proteins were incubated with IAA at varying pH, and the fraction of derivatized protein molecules was determined by mass spectrometry. Cys273 has an apparent pK_a_ of 7.4, which is lower than the 8.5 for free Cys (32) (Fig. 2*B*). The apparent pK_a_ of Cys290 is elevated at 9.7 (Fig. 2*C*).

We then asked if a disulfide link formed with CaMKII Cys273 or Cys290 is recognized by the antiserum against oxidized CaMKII. We chemically created a disulfide linkage between the purified protein and the CaMKII peptide used to generate the antiserum against oxidized CaMKII. We did this by adding a thiopyridyl leaving group to the cysteine of the CaMKII peptide in order to promote disulfide formation with the CaMKII protein (Fig. S3). HPLC/mass spectrometry confirmed that we were able to derivatize CaMKII mutants containing both Cys273 and Cys290 or only Cys273 or Cys290. All were recognized by the antiserum after incubation with the regulatory peptide–linked CaMKII (Fig. 3, *B* and *C*). As expected, the antiserum did not react with the mutant that lacked all four exposed cysteines (Fig. 3*C*). We also performed a LysC digestion and peptide purification to obtain a CaMKII peptide containing both Cys273 and Cys290 (residues 268–291). The antiserum did not recognize either the reduced peptide nor the peptide with an intramolecular disulfide between Cys273 and Cys290. We conclude that both cysteines in the CaMKII regulatory region are solvent exposed, that Cys273 is more reactive than Cys290, and that the CaMKII peptide linked to either Cys273 or Cys290 is recognized by the antiserum against oxidized CaMKII.

### Cys273 and Cys290 are necessary for CaMKII autonomous activation induced by oxidation *in vitro*

While the regulatory peptide–linked derivatives of CaMKII were recognized by the antiserum, the derivatized CaMKII did not gain autonomy, except for a very low level in the Cys273-Cys290 mutant (Fig. 3*D*). This low level may have resulted from disulfide exchange with formation of a Cys273-Cys290 linkage (see below). Since derivatization with a simple peptide does not induce autonomy, it seems that the disulfide must form between cysteine residues in the protein.

To investigate the autonomy induced by oxidative stress, we exposed CaMKII to one of the two oxidizing agents. They were diamide, which is a specific thiol oxidizing agent, and histamine chloramine, which is highly reactive with cysteines but not methionines (35). Exposure of the purified CaMKII Cys273-Cys290 mutant to diamide or histamine chloramine rendered 30% to 40% of the total activity autonomous (Fig. 3*E*). In the absence of diamide or histamine chloramine, the addition of EGTA after pretreatment with Ca^2+^/calmodulin completely abolished the activity, indicating there was no autonomy. Despite the induction of autonomy, the antiserum for oxidized CaMKII did not react with the modified CaMKII Cys273-Cys290 mutant or the WT, presumably because of insufficient exposure of the disulfide linked regions. Autonomy was reversed when the protein was incubated with DTT or thioredoxin to reduce disulfide bonds (Fig. 3*E*). When mutants lacking Cys273 or Cys290 were incubated with the oxidizing agents, no autonomous activity was observed (Fig. 3*E*).

Erickson *et al.* (24) found that binding of Ca^2+^/calmodulin was required for induction of autonomy by H_2_O_2_, and we also observed a requirement for Ca^+2^/calmodulin for induction of autonomy in the CaMKII C7S/C481S mutant exposed to diamide or histamine chloramine. This is presumably due to the requirement to expose the regulatory domain (36). It is also known that sustained activation of CaMKIIδ by Ca^2+^/calmodulin results in autophosphorylation at Thr287 and induction of autonomy because phosphoThr287 prevents the reassociation of the regulatory and kinase domains (1, 15, 37). We asked if the CaMKII autonomy induced by diamide and histamine chloramine required phosphorylation at Thr287. To answer this question, we mutated Thr287 to alanine and compared the Cys273-Thr287-Cys290 and Cys273-Ala287-Cys290 autonomous activities induced by phosphorylation (Fig. S4, *A*–*C*) or oxidation (Fig. S4, *D* and *E*). As expected, no autonomy was induced in the Cys273-Ala287-Cys290 mutant exposed to Ca^2+^/calmodulin (Fig. S4*C*), and there was no difference in the autonomy induced by diamide or histamine chloramine between Cys273-Thr287-Cys290 and Cys273-Ala287-Cys290 mutants (Fig. S4*D*). These results demonstrate that disulfide formation in the CaMKII regulatory region after pretreatment with Ca^2+^/calmodulin induces autonomy only if both Cys273 and Cys290 are present in the protein and that the autonomy induced by oxidation does not require phosphorylation of Thr287.

### An inter-dodecameric disulfide linkage between Cys273 and Cys290 renders CaMKII autonomous without Met oxidation

**Figure 4:**
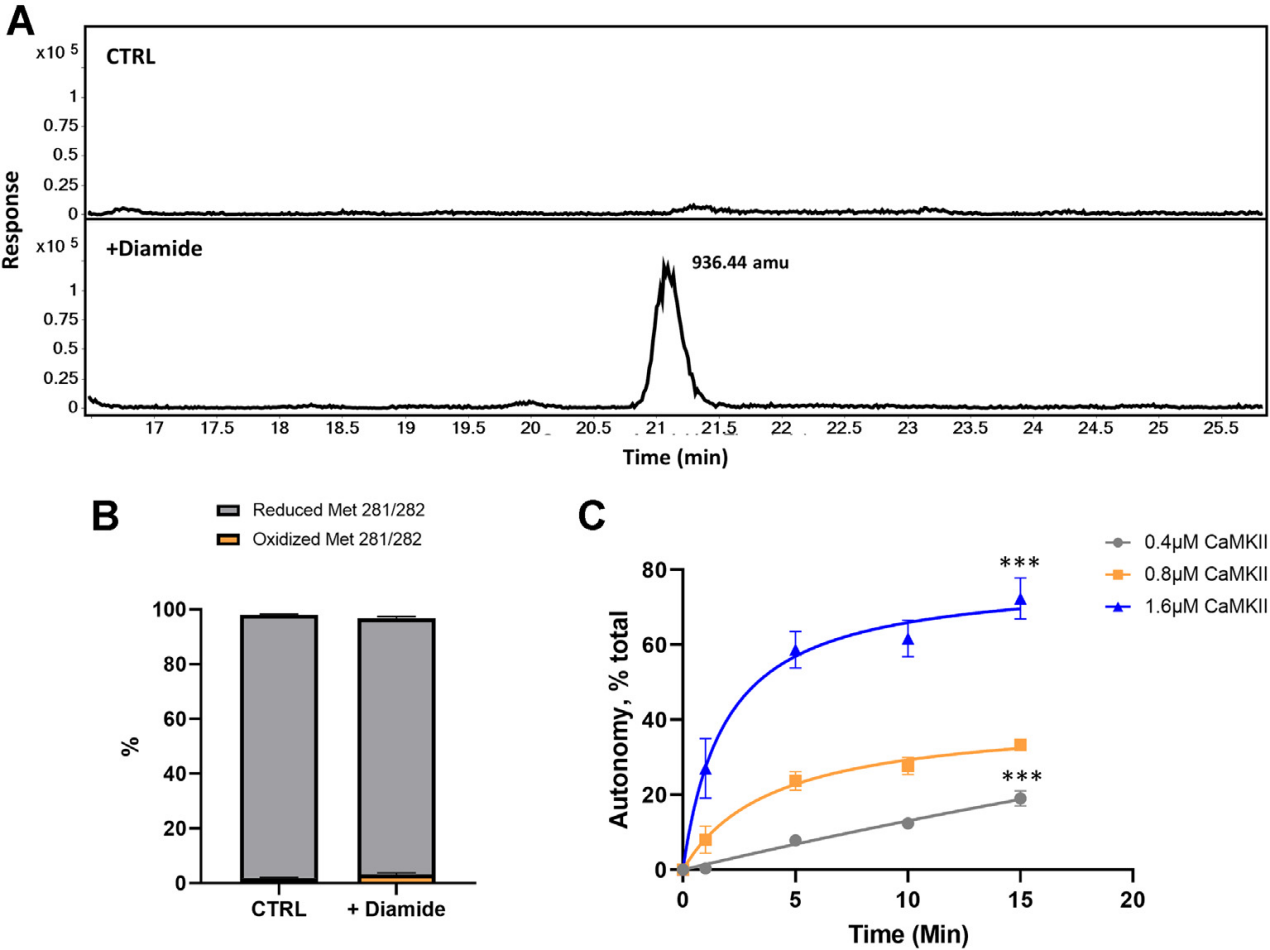
**Characterization of the disulfide formation induced by diamide.***A*, disulfide formation induced by diamide occurs between Cys273 and Cys290: CaMKII Cys273-Cys290 mutant was incubated or not with diamide and denatured and alkylated with iodoacetamide to label-free cysteine residues. After that, the enzyme was incubated with trypsin and then mapped by HPLC-MS. Only after diamide treatment (*lower panel*), two tryptic peptides were linked, residues 268 to 274 with Cys273 and residues 284 to 291 with Cys290. The calculated m/z is 936.44 amu. n = 3 replicates. *B*, methionine is not oxidized by diamide: methionine (*gray*) and methionine sulfoxide (*orange*) in the peptide containing Met281 and Met282 (residues 275–283) before and after incubation with diamide. n = 3 replicates. *C*, disulfide formation induced by diamide occurs between Cys273 and Cys290 from different CaMKII dodecamers: 0.4 μM (*gray*), 0.8 μM (*orange*), and 1.6 μM (*blue*). CaMKII Cys273-Cys290 mutant incubated with diamide during 1, 5, 10, and 15 min autonomous activity (after EGTA addition). n = 3 replicates. % of total activity (No EGTA addition). Data was fit to a hyperbola in Prism. ∗∗∗*p* < 0.01 compared with 0.8 μM CaMKII by unpaired *t* test. CaMKII, calcium/calmodulin-dependent protein kinase II.

We used HPLC-mass spectroscopy for tryptic mapping of CaMKII after oxidation with diamide and found formation of a disulfide linkage between Cys273 and Cys290 (Fig. 4*A*). The disulfide links two tryptic peptides, residues 268 to 274 containing Cys273 and residues 284 to 291 containing Cys290 (Fig. S8). All other possible dipeptides containing Cys273 were searched for in the extracted ion chromatograms, but no other was detected (Table S1). We also measured methionine and methionine sulfoxide in the peptide containing Met281 and Met282 (residues 275–283). We found 1.9% ± 0.2 methionine sulfoxide in the control and 3.2% ± 0.6 in the diamide-treated samples, a trivial difference (Fig. 4*B*).

In order to investigate if the disulfide linkage occurs only between cysteines in the same dodecamer or between different dodecamers, we incubated 0.4 μM, 0.8 μM, and 1.6 μM CaMKII with 50 μM diamide for 1, 5, 10, and 15 min and determined the dependence of the rate of oxidation on the concentration of the dodecamer. When the CaMKII concentration was doubled, the rate of oxidation increased dramatically (Fig. 4*C*). If the linkage were only between cysteines in the same dodecamer, the concentration of CaMKII would not affect the rate of oxidation. These results indicate that the disulfide formation induced by diamide between Cys273 and Cys290 from different CaMKII dodecamers contribute to autonomy. However, it does not exclude the possibility of intraholoenzyme bonds between subunits in the same dodecamer.

### Cys273 oxidation is sufficient to induce CaMKII autonomy in an HEK cell lysate

**Figure 5:**
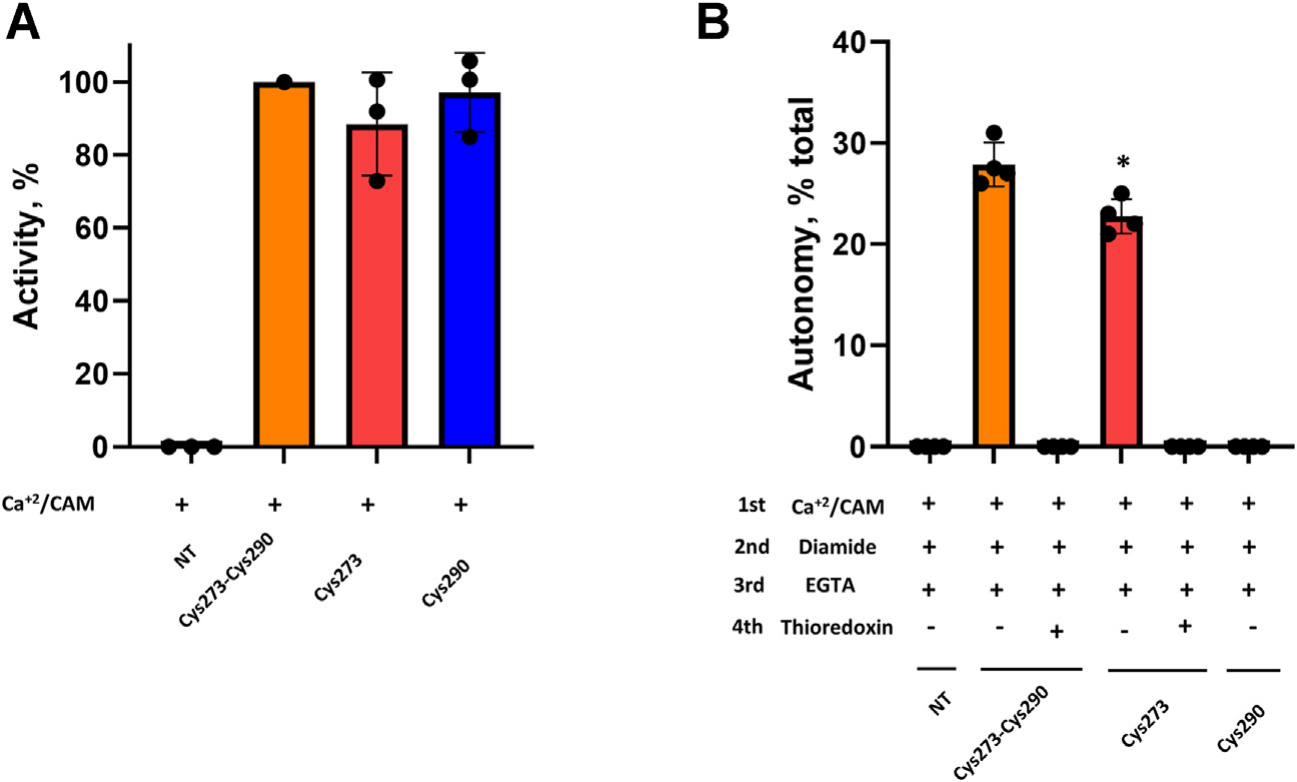
**Cys273 oxidation is sufficient to induce CaMKII autonomy in an HEK cell lysate.***A*, total activity in lysates of CaMKII Cys273-Cys290, Cys273, and Cys290 mutants expressed in HEK cells (without EGTA) compared to nontransfected HEK cells (NT). The activity of Cys273-Cys290 was set to 100%. n = 3 replicates. *B*, autonomous activity in lysates of NT, Cys273-Cys290, Cys273, and Cys290 mutants expressed in HEK cells with the lysate incubated or not with diamide, in the presence or not of thioredoxin. n = 4 replicates. ∗*p* < 0.05 compared with CYs273-Cys290 by unpaired *t* test. CaMKII, calcium/calmodulin-dependent protein kinase II.

To investigate if our results with the purified CaMKII would be reproducible with cells, we expressed CaMKII mutants containing only Cys273, only Cys290, or both in HEK293 cells. Cells were lysed, and the supernatant was used to measure CaMKII activity with or without exposure to diamide. No endogenous CaMKII activity was observed in nontransfected cells and the Ca^2+^/calmodulin-dependent activity was the same for the three mutants. (Fig. 5*A*). Autonomous activation induced by diamide was observed in the mutant containing both Cys273 and Cys290, as expected, but also in the mutant containing only Cys273. In both cases, autonomous activity was eliminated by thioredoxin (Fig. 5*B*).

Diamide-induced autonomy in the CaMKII mutant containing only Cys273 differs from that observed *in vitro* with purified proteins. With the purified enzyme, both Cys273 and Cys290 were necessary for CaMKII autonomy. However, with either purified protein or lysates, thioredoxin reversed autonomous activity, confirming that disulfide formation plays a central role in the CaMKII autonomy induced by oxidative stress. We suggest that that other cysteine-containing compounds present in the cell lysate form a disulfide bond with Cys273 with diamide exposure. Consistent with this suggestion, we found that if the purified CaMKII were incubated with GSSG; it was glutathionylated (Fig. S5*A*), and autonomy was induced in the mutant with both Cys273 and Cys290 present and in the mutant with only Cys273 present, but not when only Cys290 was present (Fig. S5*B*).

### The MMVV mutant is rapidly degraded in cell lysates

**Figure 6:**
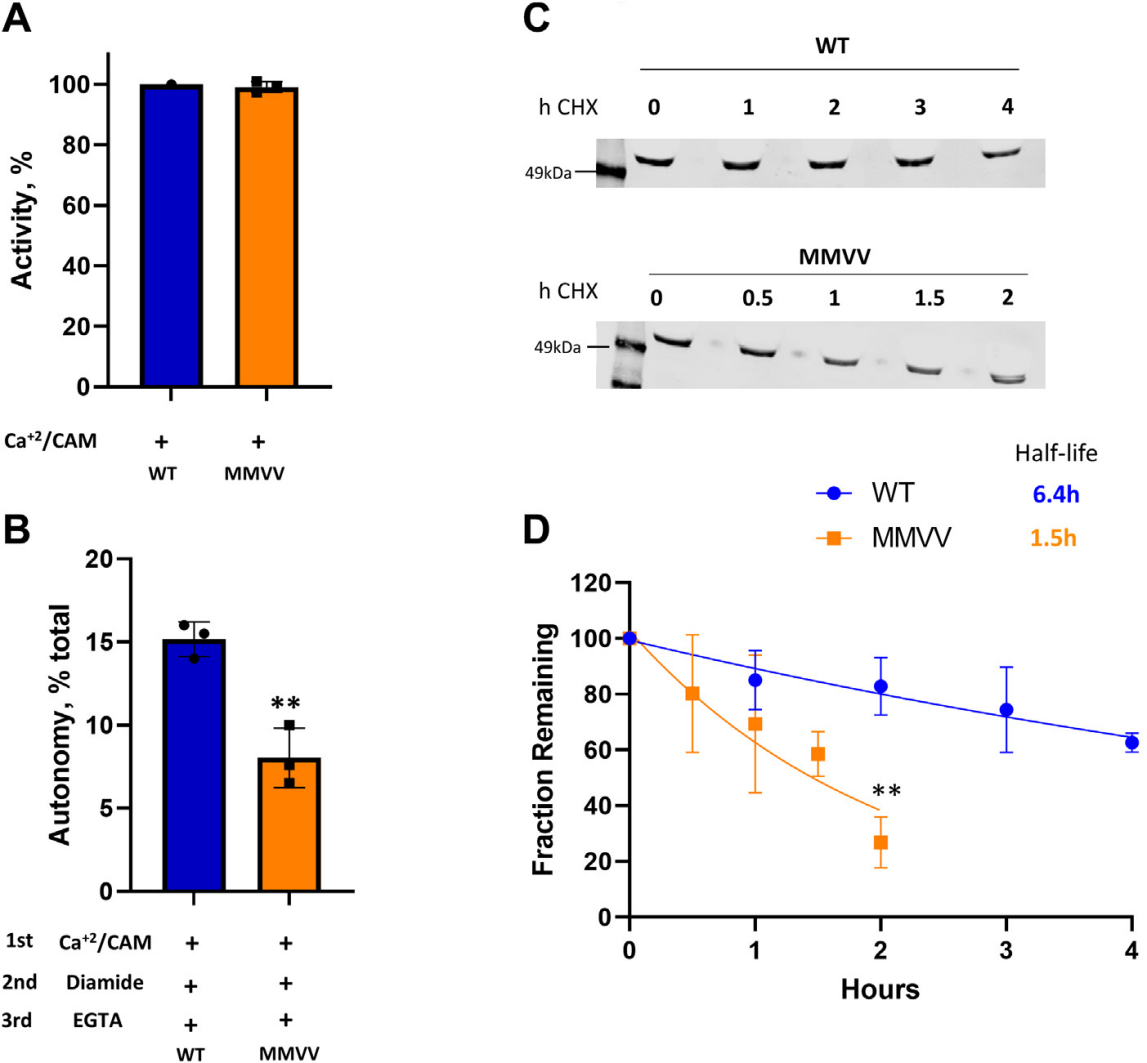
**MMVV mutant is degraded faster than the WT enzyme.***A*, purified WT and MMVV CaMKII mutants' total activity (no EGTA addition). N = 3 replicates. Normalized to WT (100%). *B*, autonomous activity of WT and MMVV CaMKII mutants treated with diamide (after EGTA addition). N = 3 replicates. % of total activity (no EGTA addition). ∗∗*p* = 0.04 compared with WT by unpaired *t* test. *C*, HEK293T cells transfected with WT or MMVV CaMKII mutants were treated with 50 μg/ml cycloheximide (CHX). Cells were lysed and the supernatant probed with anti-CaMKII antibody. Representative image of n = 3 replicates. *D*, half-life of CaMKII WT (*blue*) or MMVV mutant (*orange*). The level of CaMKII at 0 h was set to 100%. Protein loading was normalized by total protein staining. ∗∗*p* = 0.021 compared with WT by unpaired *t* test. Fit to first order decay by Prism. CaMKII, calcium/calmodulin-dependent protein kinase II.

Our results show that CaMKII autonomy induced by oxidative stress is due to disulfide formation by cysteine residues in the enzyme’s regulatory domain region and not to methionine oxidation *in vitro* as previous reported. However, the mutation of methionine residues 281 and 282 to valine, termed the MMVV mutant, was shown to protect mice and flies against cardiopulmonary pathology induced by oxidative stress (26, 38, 39). We confirmed that the WT MM and mutant MMVV CaMKII have equal activity in the presence of Ca^2+^/calmodulin (Fig. 6*A*). Based on our findings, the protection provided by the MMVV mutation could quite simply be explained if the mutation prevented disulfide formation and induction of autonomy. However, when purified WT and MMVV CaMKII were exposed to diamide, autonomy was induced in both forms, albeit to a lesser extent in MMVV than WT (∼15% for WT and 7% for MMVV, *p* = 0.04) (Fig. 6*B*). As noted above, diamide rendered autonomous ∼30% of the total activity in the C7S/C481S mutant (Fig. 3*E*). The observed quantitative differences might be explained by an increase in stability provided by the mutation of the terminus cysteines to serine.

A possible mechanism of protection against disease in various models was suggested by our observation that preparations of the MMVV CaMKII always showed aggregation and degradation, even when prepared freshly. An SDS gel with WT and MMVV mutants prepared freshly on the same day in shown in Fig. S6. This observation suggested that MMVV was unstable and might be rapidly degraded in the cell, preventing any significant accumulation of autonomous enzyme. We therefore determined the half-life of WT and MMVV CaMKII in HEK293 cells, using cycloheximide to block new protein synthesis (40). We found that the MMVV mutant is degraded ∼four times faster than the WT enzyme (Fig. 6, *C* and *D*), suggesting an unanticipated potential mechanism for the benefits of the MMVV mutant.

## Discussion

CaMKII is a multifunctional kinase with important roles in cardiovascular physiology (1, 2, 3). However, excessive CaMKII activity contributes to heart failure and arrhythmias (16, 18, 19, 20). CaMKII activity is regulated by conformational changes induced by Ca^2+^/calmodulin and posttranslational modifications. CaMKIIδ posttranslational modifications in the calmodulin-binding site have an inhibitory effect: Autophosphorylation at Thr306/307 (41, 42) and MICAL1-catalyzed oxidation of Met308 (39) inhibit CaMKII activity by diminishing Ca^2+^/calmodulin binding. In contrast, covalent modifications in the regulatory domain induce autonomous activity. Previously described modifications are autophosphorylation at Thr287 (15), *O*-GlcNAc modification at Ser280 (43), and nitrosylation at Cys290 (44). Our results establish that reaction of Cys273 and Cys290 to form a disulfide are a cause of autonomy induced by oxidative stress. Cys273 has a low pK_a_ that facilitates oxidation of its thiol to the sulfenic acid at physiological pH. The reactive sulfenic acid can then attack the thiol of Cys290 or other thiols such as GSH to form the disulfide-linked dipeptide.

We showed that the antisera widely used to detect oxidatively modified CaMKII were raised against a homodimeric dipeptide linked through a disulfide formed between the residue that is Cys273 in CaMKII. The disulfide linkage is essential for binding to the antisera, but the dipeptide needed not be a homodimer, as a randomly selected peptide linked to the CaMKII regulatory peptide is also recognized.

Increased oxidation has been shown in a variety of cardiac disease models, and generation of reactive oxygen species is known to activate CaMKII in cardiac pathologies (17, 27). Met281/282 was reported to be the target of oxidation in CaMKII that caused autonomous activity (24). However, methionine residues are notoriously susceptible to spurious oxidation (45), potentially complicating mass spectrometry analysis showing Met281/282 oxidation *in vitro* and in tissues (22, 24, 26).

Mice lacking methionine sulfoxide reductase A had increased apoptosis, cardiac dysfunction, and a higher death rate under oxidative stress than did WT mice (24), and methionine sulfoxide reductase A KO mice have increased atrial fibrillation (36) and asthma (22). These findings are consistent with the known ability of the reductase to convert methionine sulfoxide residues back to methionine (46). On one hand, methionine sulfoxide reductases provide a global antioxidant defense and thus the protective effect need not be related to CaMKII (46). On the other hand, knockin addition of MM to *Drosophila melanogaster*, where like other invertebrates VV rather than MM are present in CaMKII, causes shortened lifespan, heart tube arrhythmias, and frailty (47), suggesting the MM module is important for adverse actions of CaMKII. Intriguingly, CaMKII acquired MM at the onset of vertebrate evolution and MM has been retained through all subsequent speciation, suggesting a benefit for MM over VV. Importantly, we show that the MMVV mutant is degraded ∼ 4 times faster than the WT enzyme (Fig. 6, *C* and *D*), suggesting a previously unrecognized beneficial basis for MMVV mice and a pathological basis for MM flies, independent of methionine oxidation.

**Figure 7:**
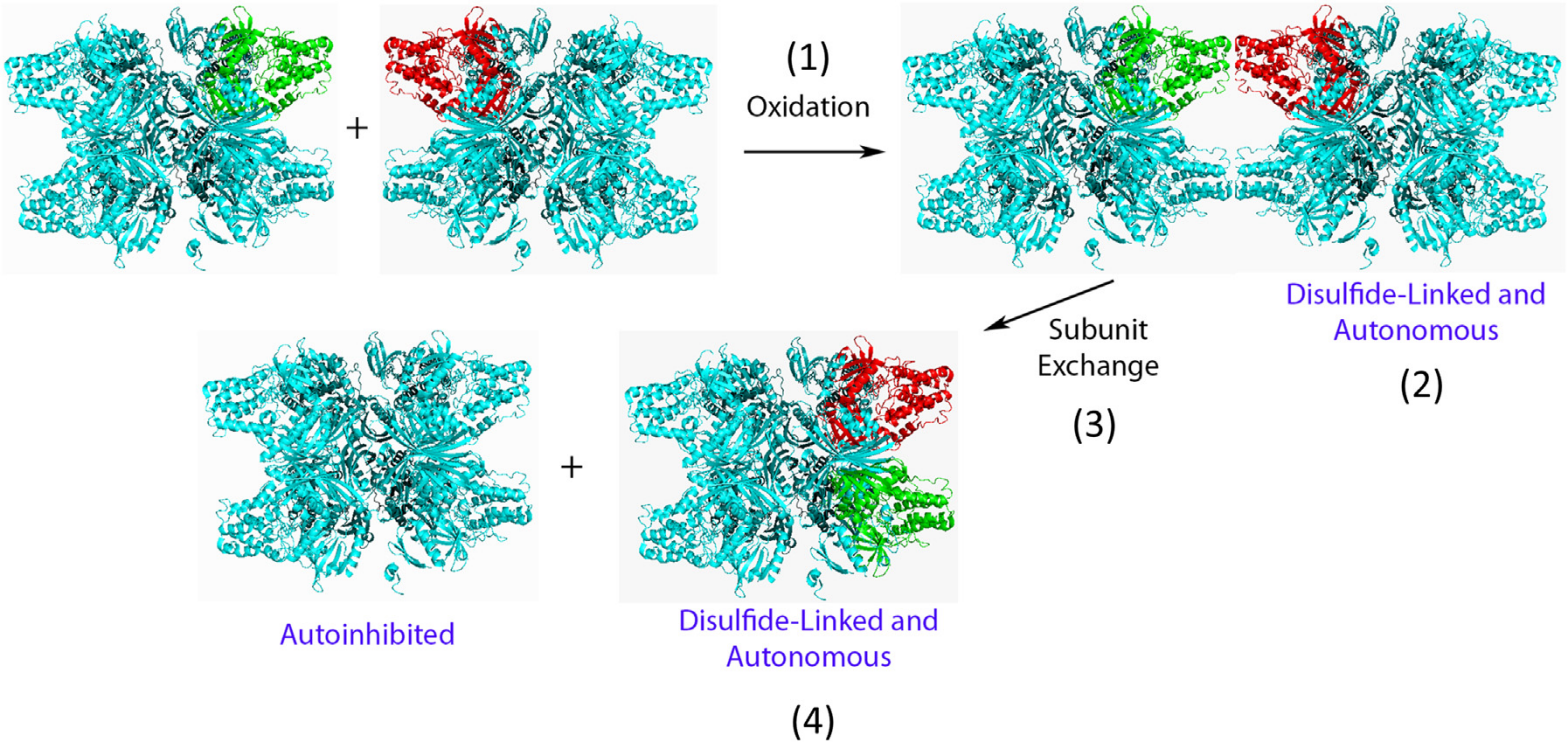
**Schematic model showing CaMKII autonomy induced by oxidation.** (1) Oxidation induces disulfide formation (2) between Cys273 and Cys290 from different CaMKII dodecamers. One subunit is colored *red* and one in a different dodecamer is colored *green*. (3) Subunit exchange occurs between the dodecamers and the disulfide is now (4) intra-dodecameric between adjacent subunits. CaMKII, calcium/calmodulin-dependent protein kinase II.

We have shown that the oxidative modification inducing autonomy in purified, recombinant CaMKII includes the formation of an inter-dodecameric disulfide between Cys273 and Cys290. Reaction between dodecamers is structurally reasonable since Cys273 and Cys290 in the same subunit are separated by 24 Å [(34), and PDB 6AYW with CaMKII alpha]. We did not observe oligomerization of dodecamers in the native gel after diamide treatment (Fig. S7). However, subunit exchange occurs readily among dodecamers (48). We suggest that the initially formed inter-dodecameric disulfide is exchanged to yield an intra-dodecameric disulfide linking adjacent subunits (Fig. 7). In any case, autonomy is reversed by exposure to thioredoxin or DTT, which are disulfide reducing agents that cannot reduce methionine sulfoxide.

Both Cys273 and Cys290 are required to induce autonomy by oxidation in the purified CaMKII. A mutant with only Cys273 did not gain autonomy, implying that the sulfenic form of Cys273 is unable to form a disulfide with Cys273 in another subunit, presumably because of lack of access. In the HEK293 cell lysate, the mutant containing only Cys273 did gain autonomy when exposed to diamide, presumably by reaction with other Cys-containing peptides or proteins in the lysate. Consistent with this suggestion, purified CaMKII that was glutathionylated at Cys273 does gain autonomy.

The structural basis for the increased turnover of MMVV CaMKIIδ has not yet been investigated by us. However, Valley *et al.*, 2012 (49) established that methionine commonly forms a noncovalent bond with the ring of aromatic amino acids, and this interaction provides stabilization of the protein. The distance between the bonded sulfur of methionine and the aromatic ring is typically 5 to 7 Å (49). In CaMKII, the distance between Met281 and Tyr107 is 6.2 Å [(34) and PDB 2VN9]. Mutation of Met281 to Val would abolish this interaction and might induce a conformational change that leads to rapid degradation. The protection provided by the MMVV mutation informs us that accelerating the turnover of CaMKII may ameliorate or prevent the deleterious effects of chronic autonomous activation, and targeted protein degradation is an emerging therapeutic modality (50, 51).

## Experimental procedures

### Calculation of cysteine and methionine oxidation rates

The calculation of the cysteine and methionine oxidation rates was from the second order rate equation: ([B][A]_0_/[A][B]_0_) = k([B]_0_−[A]_0_)t. B_0_ = initial H_2_O_2_ concentration, B = final H_2_O_2_ concentration, A_0_ = initial concentration of cysteine or methionine, A = final concentration of cysteine or methionine, and t = time in hours. The second order rate constant, k, of cysteine is 61,200 M^−1^ h^−1^ and of methionine, 32.07 M^−1^ h^−1^ (32, 33).

### Immunoprecipitation assay

CaMKII peptide, CQRSTVASMMHRQETVD, was synthesized by Bacham who also supplied an unrelated control peptide with an amino terminal Cys, CGHGNKSGLMVGGVV (Cat #: H6364). The peptide was dissolved in buffer, 50 mM Hepes, 1 mM DPTA, pH 7.5, to give 100 μM peptide. To form the disulfide-linked dimer, the solution was made 1 mM in H_2_O_2,_ incubated at 37 °C for 15 min, and an aliquot was taken. To produce the disulfide-linked dimer with Met oxidized to MetO, the solution was brought to 10 mM H_2_O_2,_ incubated for another 15 min, and another aliquot was taken. To produce the monomeric peptide with MetO, the solution was incubated with 10 mM DTT for 15 min, and the final aliquot was taken. The aliquots were purified and analyzed by HPLC-mass spectrometry as described below. The mass of each purified peptide was within 0.5 Da of that calculated from its sequence.

Protein A Sepharose (GE Healthcare, Cat #: 17-0780-01) was prepared following the vendor’s instructions: 40 μg of beads were incubated with 10 μg of ox-CaMKII antibody (Genetex, Cat#: GTX111401) in PBS for 2 h at 4 °C. Beads were then washed 3 times with IP buffer (50 mM Hepes, 1 mM DPTA pH 7.5). Twenty micromolars of each peptide were separately incubated with the beads in IP buffer overnight. The beads were then washed with IP buffer three times. They were eluted with 200 mM glycine, pH 2.6 for 10 min with frequent agitation before gentle centrifugation. The eluate was neutralized with Tris and was analyzed by HPLC-mass spectrometry as described below.

### Effect of incubating the CaMKII peptide with a low concentration of H_2_O_2_

To mimic the oxidation conditions employed by Erickson *et al.* (24), CaMKII peptide, CQRSTVASMMHRQETVD, was dissolved in 0.05% TFA to make a 5 mM stock, and an aliquot was diluted to 10 μM with buffer (50 mM Hepes, 1 mM DTPA, pH 7.5), with a sample taken for analysis. Then the solution was made 100 μM in H_2_O_2_, incubated at 37 °C 1 h, and again a sample was taken for analysis. The solution was then made 10 mM in DTT, incubated for another 10 min, and sampled again. Samples from each of the three steps (before H_2_O_2_ treatment, after H_2_O_2_ treatment, and after DTT treatment) were analyzed by HPLC-mass spectrometry as described below.

### CaMKIIδ expression by transient transfection of tsA201 cells

Recombinant human CaMKIIδ (GenBank #NP 001308500) was cloned into the eukaryotic expression vector pLEXm (52) (forward primer: 5′ ggccgaattcgaatggcttcgaccaccacctgcacccgg-3′ and reverse primer 5′ tccagggtacctcagatgttttgcc-3′). Mutant CaMKII cDNAs were generated using a QuikChange site-directed mutagenesis kit (Agilent).

tsA201 cells derived from HEK293 (MilliporeSigma, 96121229-1VL) were adapted to grow in suspension by using Gibco FreeStyle 293 Expression Medium plus 2% fetal bovine serum, 50 U/ml penicillin-streptomycin, and 2 mM glutamine in a shake flask. A low passage (P2 or P3) of the suspension culture was collected as the cell stock. It was made to 7.5% in dimethyl sulfoxide and aliquots were stored in liquid nitrogen. For each expression, one frozen vial with 1 × 10^7^ tsA201 cells was inoculated into 40 ml of the same medium in a 250 ml flat bottom cell culture shaker flask. Cells were grown for 3 days in Infors Multitron incubator at 130 rpm with 8% CO_2_ and 75% humidity. Cell number was measured with a NucleoCounter NC-100. Cells were then passed into a large flask (2 l or 3 l Fernbach Flask, Vent Cap, Plain Bottom) with addition of the above medium to give a final cell density of 1.3 × 10^5^/ml. The total volume of culture was no more than 0.5 l for the 2 l shake flask and no more than 1 l for the 3 l flask. Transfection was initiated when cells reached a density of 2 × 10^6^/ml, usually after 3 days growth. For the 1 l culture, 1 mg of pLEXm-CaMKII plasmid (filtered with 0.22 μm filter) was added to 50 ml prewarmed Hybridoma-SFM (ThemoFisher, 12045076). Then, 4 ml of PEI MAX (Transfection Grade Linear Polyethylenimine Hydrochloride, MW 40,000, Polysciences, 24765-1) at 1 mg/ml was added and gently mixed to make it homogenous. After incubation at room temperature for 12 to 15 min, the solution was added to the culture and the flask returned to the incubator. After 3 days, cells were pelleted by centrifugation at 300*g* for 15 min in a Sorvall RC3B plus centrifuge. Cell pellets were washed once with 1× PBS and stored at −80 °C until used.

### CaMKIIδ purification

Cells were resuspended in homogenization buffer (50 mM Tris–HCl pH 7.5, 150 mM NaCl, 1 mM PMSF (MilliporeSigma, P7626), a protease inhibitor cocktail (MilliporeSigma, P2714)) and sonicated to release the cell contents. The lysate was then centrifuged at 12,000*g* at 4 °C for 20 min. The supernatant was removed and added to three volumes of equilibration buffer (50 mM Tris–HCl pH 7.5, 150 mM NaCl, 2 mM CaCl_2_). CaM-agarose (BioVision, 7934-10) was prepared by washing three times with wash buffer (50 mM Tris–HCl pH 7.5, 100 mM NaCl, 1 mM CaCl_2_). CaMKII was eluted from the resin in five fractions of 1 ml elution buffer (50 mM Hepes pH 7.5, 150 mM NaCl, 10 mM EGTA). Protein fractions were incubated with 10 mM DTT for 30 min and dialyzed overnight at 4 °C into storage buffer (50 mM Hepes pH 7.5, 150 mM NaCl, 5 mM DTPA). The fractions containing CaMKII protein were identified by SDS-PAGE, and the CaMKII mass was verified by HPLC-mass spectrometry to be within 0.5 Da of that calculated from its sequence.

We found that the kinase produced in these mammalian cells differs from that produced in baculovirus, which we and others have previously used for production of CaMKII. The HEK293 cell product must have a different conformation from that from baculovirus because the mammalian enzyme is much more stable and has a substantially higher specific activity. The enzyme produced by baculovirus typically requires very high concentrations of glycerol to stabilize it (24, 39), while that from HEK293 cells was stable for at least a month at 0 °C in a simple Hepes buffer. The typical specific activity of baculoviral CaMKII is ∼60 nmol/min/mg (24, 39), while that from our CaMKII purified from HEK293 cells is ∼9.5 μmol/min/mg.

### HPLC-mass spectrometry

Protein separations and mass determinations were performed on a Zorbax 300 Å StableBond C18 MicroBore column (1.0 × 50 mm, 3.5 μm particle size, Agilent 865630-902) with an Agilent 1200 series high pressure liquid chromatography system with the column compartment set to 30 °C. The initial solvent was water/0.05% TFA and proteins were eluted by a gradient of 2%/min acetonitrile/0.05% TFA with a flow rate of 50 μl/min. Effluent from the column was mixed in a tee with 5 μl/min neat acetic acid just prior to the electrospray needle to displace the bound TFA and to generate internal standards (53, 54).

### CaMKIIδ expression by transient transfection of HEK293 cells

HEK293 cells were cultured in Dulbecco’s modified Eagle’s medium (Invitrogen 11965092) with 10% fetal calf serum, and 1% penicillin and streptomycin at 37 °C in a humidified atmosphere of 5% CO2 and 95% air. Cells were transfected using calcium phosphate (Invitrogen K278001). Forty eight hours after transfection, cells were lysed with 25 mM Tris–HCl, pH 7.4, 150 mM NaCl, 1 mM EDTA, 1% Nonidet P-40, 5% glycerol, 1 mM PMSF, and 1× protease inhibitor cocktail. Lysates were cleared by centrifugation for 20 min at 20,800*g* at 4 °C. Western blot or native PAGE analysis and the CaMKII activity assay were performed as described below.

### CaMKII activity assay

For all assays, the incubation solution was 50 mM Hepes, pH 7.4, 10 mM MgCl_2_, 500 μM Ca^2+^, 1 mg/ml bovine serum albumin, 1 μM CaM, 400 μM ATP. To measure the maximal specific activity, purified CaMKII (1.6 ng protein) was incubated with 50 μM syntide 2 (MilliporeSigma, SCP0250) for 30 min at 37 °C. For all other assays of purified protein, 0.35 ng CaMKII was incubated with 6 μM syntide for 10 min at 37 °C in the same assay mixture. To measure CaMKII activity in the HEK293 cells, 30 μg of the supernatant was incubated with 6 μM syntide for 10 min at 37 °C. Reactions were stopped by the addition of 1 μl 10% TFA.

Phosphorylated and nonphosphorylated peptides were separated with the HPLC system and column described above for HPLC-mass spectrometry except that the acetonitrile gradient was developed as: 0 to 5%, 0 to 1 min; 5 to 16.5%, 1 to 24 min; 16.5 to 95%, 24 to 32 min; and 95 to 0%, 32.0 to 32.1 min. The fraction of phosphorylated peptide was calculated from the integrated areas of the peaks in the UV chromatogram at 210 nm.

### CaMKII oxidation

To prepare histamine chloramine, HOCl (MilliporeSigma) in PBS was added dropwise with rigorous mixing to histamine (MilliporeSigma) at a molar ratio of 1:1 (35, 55). 0.8 μM purified CaMKII was incubated with 0.8 μM histamine chloramine for 15 min or 50 μM diamide (MilliporeSigma) for 5 min at 25 °C. CaMKII-transfected HEK293 cell supernatant was incubated with 50 μM diamide (MilliporeSigma) for 5 min at 25 °C. Histamine chloramine induced maximal autonomy very quickly while diamide was slower (Fig. S9).

To measure CaMKII autonomy (activity after Ca^2+^/CAM chelation), the purified protein or HEK293 cells supernatant was incubated with 500 μM Ca^2+^ and 1 μM CAM in 50 mM Hepes buffer for 5 min, with or without diamide (additional 5 min) or histamine chloramine (additional 15 min) followed by 10 min incubation with 10 mM EGTA at 25 °C. In some assays, 0.4 μM thioredoxin or 10 mM DTT were added after the oxidants for 10 min. After that, the protein was incubated with 6 μM syntide for 10 min at 37 °C, and the phosphorylated and nonphosphorylated peptides were separated as described above.

### Synthesis of thiopyridylated-CaMKII peptide and CaMKII derivatization

2,2′-dithiodipyridine was purchased from MilliporeSigma (D5767). A 100 μM solution of the CaMKII peptide, CQRSTVASMMHRQETVD (residues 273–299), was prepared in 20% acetic acid. A 10 mM solution of dithiodipyridine was made in methanol. In a glass tube, 100 μl peptide was pipetted, then 5 μl dithiodipyridine, and the solution was mixed by vortexing. The reaction is complete within 2 min, as determined by HPLC-mass spectrometry. For extraction, 200 μl ether was added, mixed, and the tube centrifuged briefly to assure phase separation. The upper ether layer was removed and discarded. The ether extraction was repeated three more times. Then the derivatized peptide was aliquoted, the tubes dried in a vacuum centrifuge, and then stored at −80 °C until use. We used 0.05% TFA to redissolve the thiopyridylated-peptide, as it is poorly soluble in the buffer used for the CaMKII proteins. The dithiopyridone reagent has a peak at 281 nm with ƐM = 9730 (56). One may use half that value, ƐM = 4865, for quantitation of the thiopyridylated-peptide.

For reaction with the protein, 1 μM CaMKII was incubated with 500 μM Ca^2+^, 1 μM CaM, and 6 μM thiopyridylated-CaMKII peptide for 10 min at 25 °C. The product, regulatory peptide–linked CaMKII was quantitated by HPLC–mass spectrometry.

### CaMKII glutathionylation

Glutathionylation was carried out by disulfide exchange with GSSG (MilliporeSigma). One micromolar CaMKII was incubated with 5 mM GSSG for 1 h at 37 °C. The reaction was stopped by making it 5% in acetic acid. Monoglutathionylation and diglutathionylation were quantitated by HPLC–mass spectrometry.

### SDS-page and Western blot

HEK293 supernatant or purified CaMKII protein concentration was determined by the BCA method (Thermo Fisher Scientific). Protein samples were mixed with 2× Novex Tris-glycine SDS sample buffer (Invitrogen) supplemented with 1 mM 2-mercaptoethanol and heated for 3 min at 100 °C when using the primary antibody recognizing CaMKII delta (GeneTex - 1:10,000). 2-mercaptoethanol was not added when using the primary antibody recognizing CaMKII oxidized (GeneTex - 1:1000) or CaMKII beta/gamma/delta phosphoThr287 (GeneTex - 1:10,000).

The same amount of protein (20 μg for supernatant and 1 μg for purified protein) was loaded onto each lane, and SDS-PAGE was performed on Novex 10 to 20% Tris-glycine gel in Novex Tris-glycine SDS running buffer (Invitrogen) at maximal 125 V for 2 h. Then, proteins were transferred to 0.45-μm pore size nitrocellulose membranes (Invitrogen) at 70 V for 3 h at 4 °C using a Bio-Rad Tank Transfer System. Membranes were blocked for 30 min with blocking buffer (Li-Cor), followed by incubation in blocking buffer supplemented with 0.05% Tween 20 and 1:10,000 diluted primary antibodies overnight on a rocking platform at 4 °C. The next day, incubation was continued for 1 more h at room temperature and then washed with 1× PBS, pH 7.4, twice for 10 min, followed by incubation in blocking buffer (0.05% Tween 20) supplemented with a 1:25,000 dilution of Alexa Fluor 680-conjugated secondary antibody (Invitrogen) for 20 min at room temperature on a rocking platform. Membranes were then washed with PBS for 15 min with a change of buffer every 5 min. Quantitative Western blot images were obtained by scanning the membrane on a Li-Cor Odyssey infrared imaging system according to the manufacturer’s instructions. Protein loading was normalized by total protein staining (Revert Total Protein Stain Li-Cor).

### Native-PAGE

HEK293 supernatant or purified CaMKII protein concentration was determined by the BCA method. Protein samples were mixed with 4× NativePAGE Sample Buffer (Thermo Fisher Scientific), NativePAGE 5% G-250 Sample (Thermo Fisher Scientific), and water for a final volume of 10 μl. The same amount of protein (20 μg for supernatant and 1 μg for purified protein) was loaded onto each lane, and Native-PAGE was performed on NativePAGE Novex 3 to 12% Bis-Tris gel. The gel was run with cathode buffer (1× NativePAGE Running Buffer, 1× NativePAGE Cathode Additive) and anode buffer (1× NativePAGE Running Buffer) at maximal 150 V for 2 h. The gel was stained with Coomassie Brilliant Blue R-250 (Bio-Rad) and scanned on a Li-Cor Odyssey infrared imaging system according to the manufacturer’s instructions.

### CaMKII tryptic mapping

For the double alkylation (Fig. 2*A*), purified CaMKII was first alkylated with IAA to label exposed cysteine residues. Then the protein was denatured and disulfide bonds were reduced. The newly available reduced thiols were then labeled with acrylamide. Those cysteines that were exposed in the native protein had a mass increase of 57.1 Da by reaction with IAA while those that were in disulfide linkage had an increase of 71.0 Da by reaction with acrylamide. These results allowed calculation of the exposed fraction of each cysteine-containing peptide. In detail, 5 μg CaMKII was incubated with 10 mM IAA in the dark for 30 min at 25 °C. The reaction was stopped with an equal volume of 20% trichloroacetic acid (TCA) for 5 min on ice and centrifuged for 5 min at 16,000*g*. The supernatant was discarded and the pellet-containing protein and residual TCA was extracted twice with 0.1 ml ethanol:ethyl acetate (1:1 v/v), centrifuging 5 min at 16,000*g* after each extraction. After air drying, the protein was resuspended in denaturing buffer (6 M guanidine, 100 mM Tris, 1 mM DTPA, pH 8.5) and incubated with 5 mM DTT at 25 °C for 30 min. After that, 50 mM acrylamide was added and incubated for 30 min at 37 °C. The reaction was stopped with an equal volume of 20% TCA for 5 min on ice and centrifuged for 5 min at 16,000*g*. The supernatant was discarded and the pellet-containing protein and residual TCA was extracted twice with 0.1 ml ethanol:ethyl acetate (1:1 v/v), centrifuging 5 min at 16,000*g* after each extraction. After air drying, the protein was resuspended in trypsinization buffer (100 mM Tris, 1 mM DTPA, pH 8.5) and subjected to tryptic digestion overnight at 37 °C with a ratio of protease:CaMKII of 1:20 (Promega, V511A). The reaction was stopped by making the solution 0.1% in TFA. Peptides were sequenced by HPLC-mass spectrometry.

For the single alkylation to determine thiol status (Fig. 4*A*), purified CaMKII was incubated with diamide, denatured, and alkylated with IAA to label thiol-containing cysteine residues. Five micrograms CaMKII was incubated with 50 mM diamide for 5 min at 25 °C. The reaction was stopped with an equal volume of 20% TCA for 5 min on ice and centrifuged for 5 min at 16,000*g*. The supernatant was discarded and the pellet that contained protein, residual TCA, and precipitated guanidine was extracted twice with 0.1 ml ethanol:ethyl acetate (1:1 v/v), centrifuging 5 min at 16,000*g* after each extraction. After air drying, the protein was resuspended in denaturing buffer (6 M guanidine, 100 mM Tris, 1 mM DTPA, pH 8.5). After that, 10 mM IAA was added and incubated in the dark for 30 min at 25 °C. The reaction was stopped with an equal volume of 20% TCA for 5 min on ice and centrifuged for 5 min at 16,000*g*. The supernatant was discarded and the pellet-containing protein and residual TCA was extracted twice with 0.1 ml ethanol:ethyl acetate (1:1 v/v), centrifuging 5 min at 16,000*g* after each extraction. After air drying, the protein was resuspended in trypsinization buffer (100 mM Tris, 1 mM DTPA, pH 8.5) and subjected to tryptic digestion overnight at 37 °C with a ratio of protease:CaMKII of 1:20. The reaction was stopped by making the solution 0.1% in TFA. Peptides were sequenced by HPLC-mass spectrometry.

Peptide separation and sequencing was carried out as for proteins except that the gradient was developed as follows: 0 to 40%, 0 to 40 min; 40 to 100%, 40 to 45 min; and 100 to 0%, 46 to 47 min. Electrospray mass spectrometry was performed on an Agilent Model 6520 accurate mass quadrupole-time of flight instrument. Positive electrospray ionization spectra were obtained in the mass range of 100 to 2500 m/z. The drying gas temperature was 350 °C with a flow rate of 10 l/min and a nebulizer pressure of 2 bar. The voltages were capillary 3500 V, fragmentor 235 V, skimmer 65 V, and octopole 1 750 V. MS/MS fragmentation used a collision energy of 30 V with a data collection range of 20 to 2000 m/z. Mass spectra were analyzed using Agilent software, MassHunter version B.07. Predicted MS/MS spectra were generated by GPMAW and matched to the experimentally obtained spectra.

### CaMKII LysC digestion

1 mg/μl solid guanidine (final concentration 6 M) was added to 5 μg purified CaMKII and incubated with 10 mM DTT at 37 °C for 30 min. Then the tube was placed on ice for 10 min and an equal volume of 20% TCA was added. The tube was placed on ice again for 10 min and centrifuged for 3 min at 20,200*g*. The supernatant was discarded and 50 μl of 100% dimethylformamide was added. After vortexing, the sample was centrifuged for 3 min at 20,200*g*, and the supernatant was discarded. This step was repeated twice. One hundred microliters of 100% ether was added to remove the residual dimethylformamide. The sample was centrifuged for 3 min at 20,200*g* and the supernatant was discarded. This step was repeated twice. After air drying, the protein was resuspended in buffer (100 mM Tris, 1 mM DTPA, pH 8.5) and subjected to LysC digestion overnight at 37 °C with a ratio of protease:CaMKII of 1:20 (Wako, 121-05063). The reaction was stopped by making the solution 0.1% in TFA. Peptides were sequenced by HPLC-mass spectrometry.

Peptide separation and sequencing was carried out as described above except that the gradient was developed as follows: 0 to 15%, 0 to 3 min; 15 to 30%, 3 to 33 min; 30 to 95%, 33 to 46 min. The reduced and oxidized peptide P268-291 was purified by HPLC reverse phase.

## Data availability

Data generated in this study are included in the article or supporting information.

## Supporting information

This article contains supporting information.

## Conflict of interest

M. E. A. has a financial interest in CaMKII inhibitor molecules owned by Johns Hopkins University.
