# Supplementary material for: Oxidative stress–induced autonomous activation of the calcium/calmodulin-dependent kinase II involves disulfide formation in the regulatory domain
Source: J Biol Chem. 2022 Oct 8;298(11):102579. doi: 10.1016/j.jbc.2022.102579 (PMC9643438; doi:10.1016/j.jbc.2022.102579)
Supplement: Supplemental Figures S1–S9 and Table S1 [file mmc1.pdf]

Supporting information

Oxidative stress-induced autonomous activation of the Calcium/Calmodulin-Dependent Kinase II involves disulfide formation in the regulatory domain

Nathália Rocco-Machado[1], Lo Lai[1], Geumsoo Kim[1], Yi He[2], Elizabeth D. Luczak[3], Mark E. Anderson[3,4,5], Rodney L. Levine[1]*

[1] Laboratory of Biochemistry, National Heart, Lung and Blood Institute, Maryland, United States of America, [2] Fermentation Facility, National Heart, Lung, and Blood Institute, National Institutes of Health, Maryland, United States of America. [3] Division of Cardiology, Department of Medicine, Johns Hopkins University, Baltimore, United States of America, [4]Department of Physiology and Program in Cellular and Molecular Medicine, The Johns Hopkins University School of Medicine, Baltimore, MD 21205, USA, [5]Department of Genetic Medicine, The Johns Hopkins University School of Medicine, Baltimore, MD 21205, USA

*Corresponding author E-mail: rlevine@nih.gov

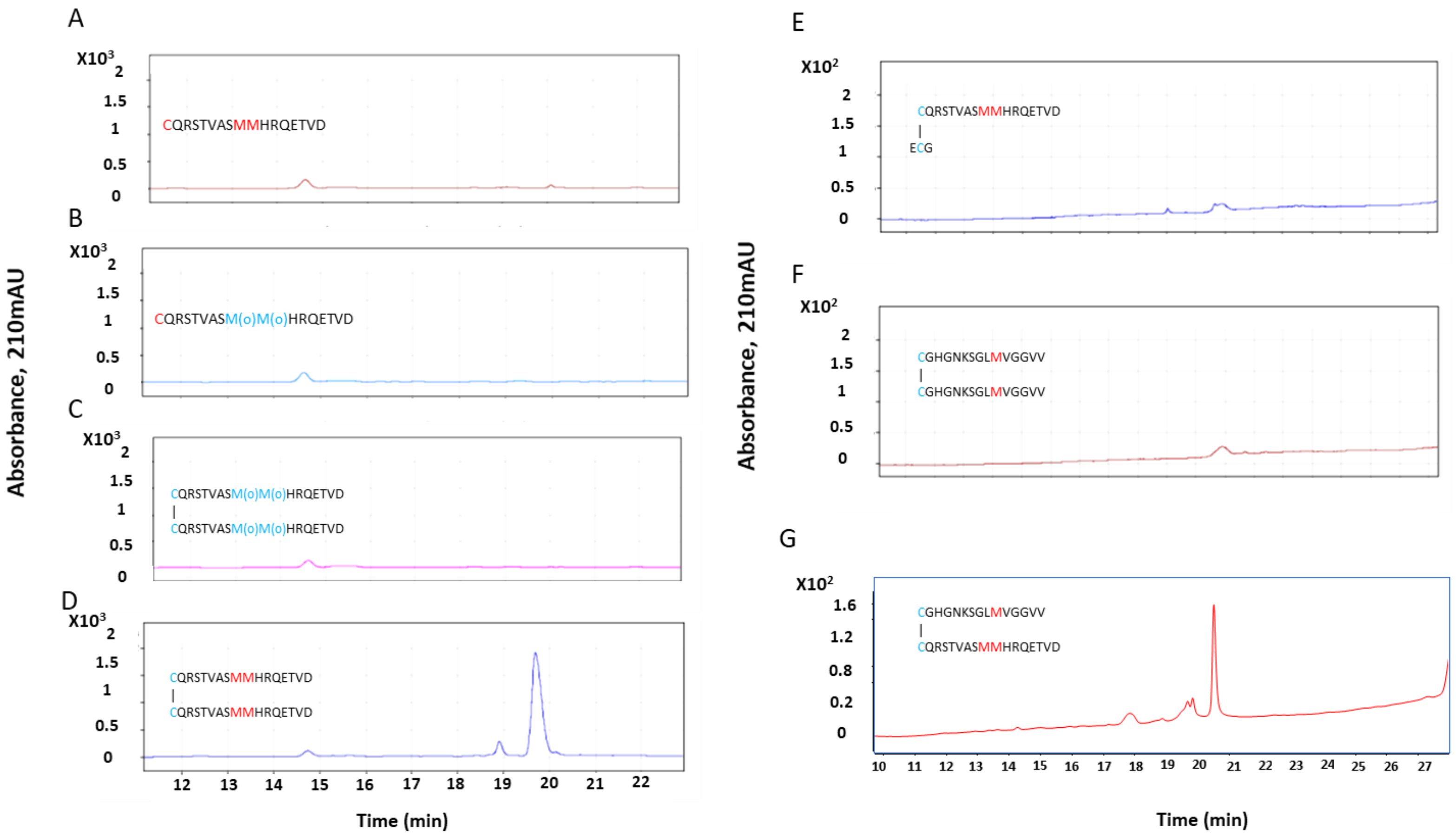

Supplementary figure 1: The antiserum raised against the H_2O_2-exposed peptide detects disulfide-containing CaMKII and not Met281/Met282 sulfoxide. Protein A magnetic beads were coupled with ox-CaMKII antibody and incubated with the different peptide samples, as indicated in the figure. Their binding was determined by HPLC-mass spectrometry (representative image of n=2 replicates).

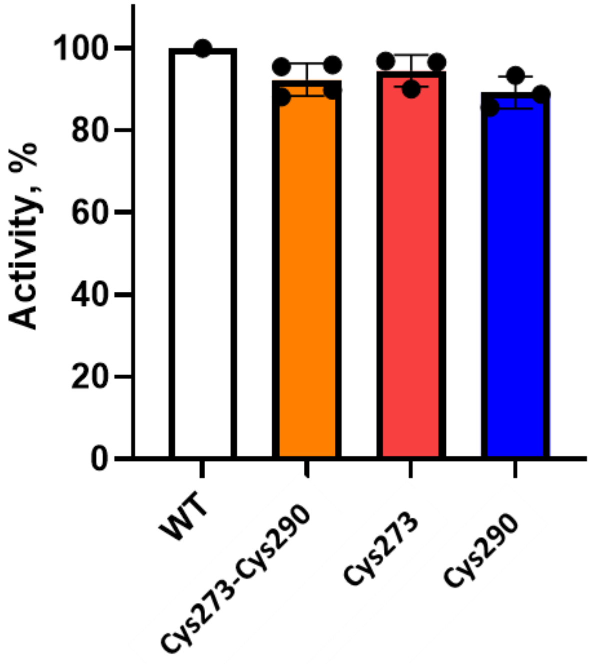

Supplementary figure 2: No difference between Wild Type and CaMKII mutants' total activity. n=3 replicates. Normalized to 100% for the Wild Type.

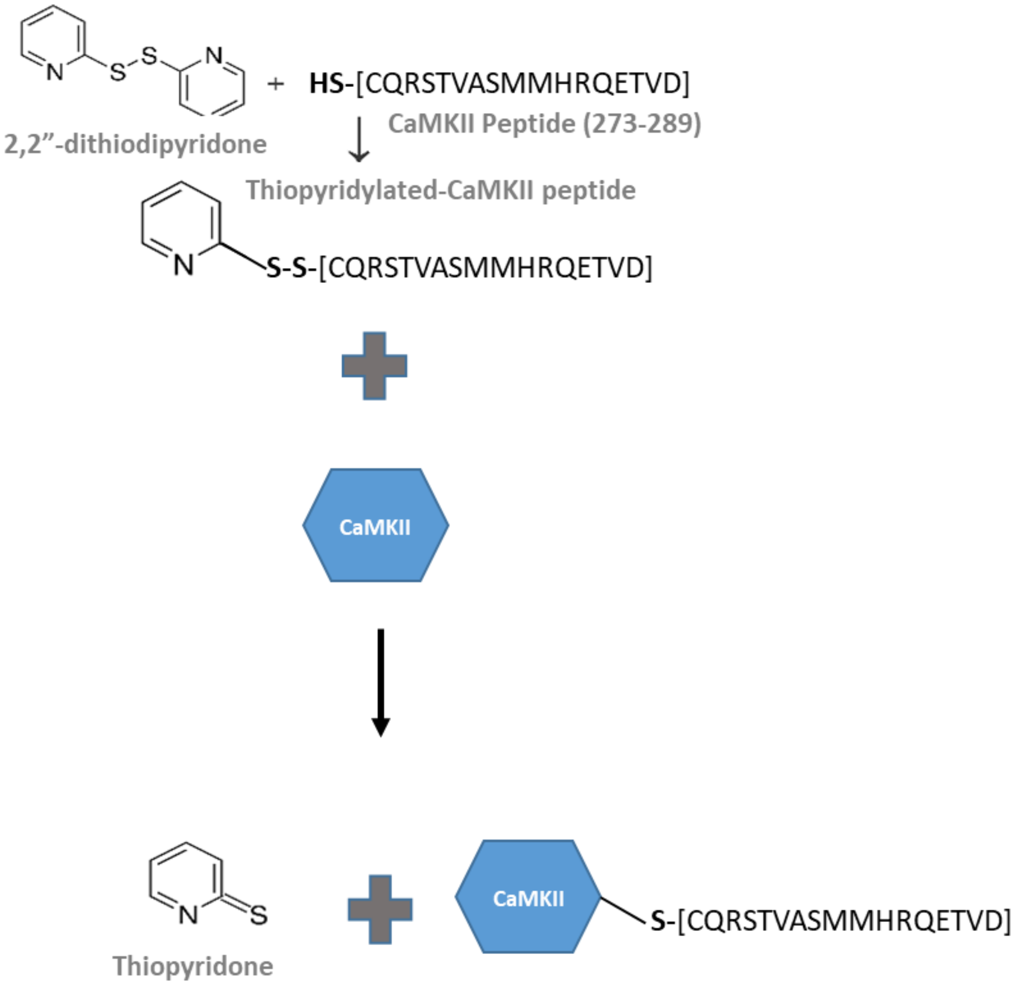

Supplementary figure 3: Synthesis of thiopyridylated-CaMKII peptide and CaMKII derivatization.

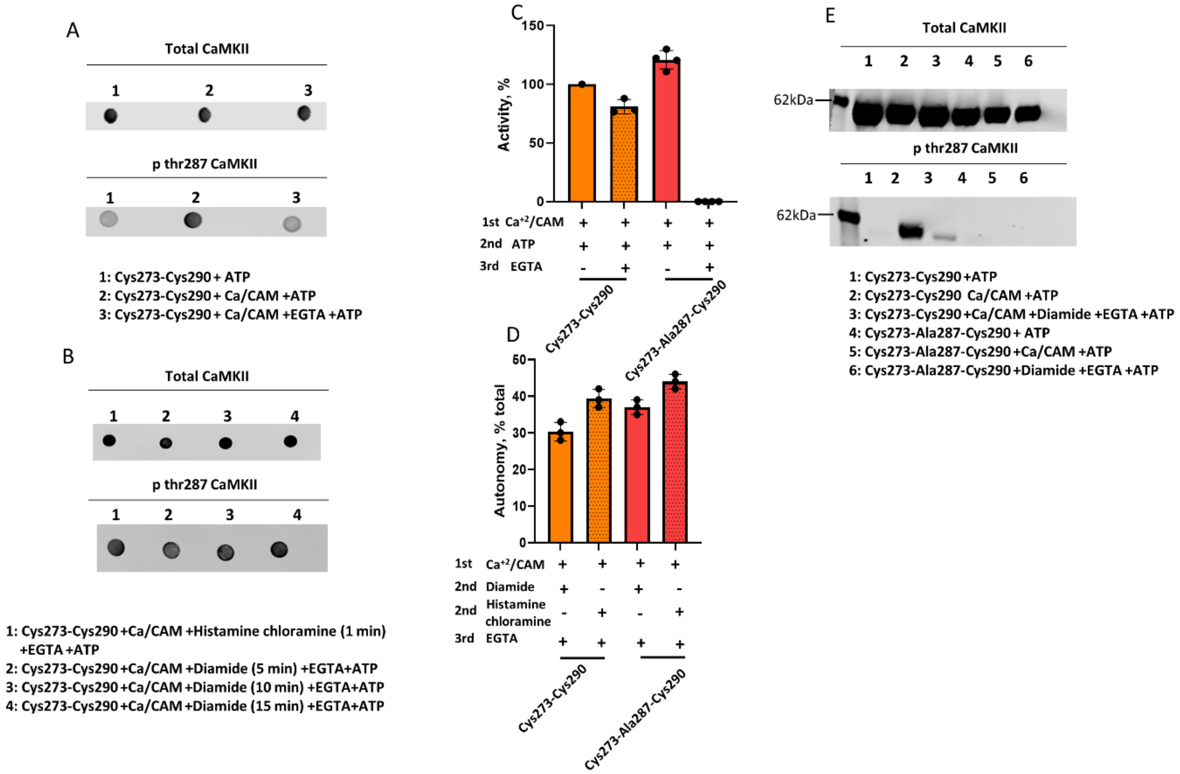

Supplementary figure 4: Autonomy induced by diamide does not depend on Thr287 phosphorylation. (A) Dot Blot - Purified CaMKII Cys273-Cys290 incubated with ATP, Ca^{+2}/CAM + ATP or Ca^{+2}/CAM + EGTA + ATP was probed with anti-CaMKII antibody or anti-Thr287 phosphorylated antibody (representative image of n=3 replicates). As expected, the sample was only recognized by the anti-Thr287 phosphorylated antibody when incubated with Ca^{+2}/CAM and ATP. When ATP was added after Ca^{+2}/CAM chelation by EGTA no phosphorylation was observed. **(B)** However, when Cys273-Cys290 was incubated with Ca^{+2}/CAM and histamine chloramine (1min) or diamide (5, 10 or 15min), the phosphorylation of Thr287 occurred even after Ca^{+2}/CAM chelation. This result indicates that disulfide formation keeps the CaMKII catalytic site open even in the absence of Ca^{+2}/CAM. **(C)** Purified CaMKII Cys273-Cys290 and Cys273-Ala287-Cys273 total activity (without EGTA) and pThr287 induced autonomous activity (after EGTA addition). N=4 replicates. Normalized to Cys273-Cys290 at 100%. **(D)** Purified CaMKII Cys273-Cys290 and Cys273-Ala287-Cys290 oxidation induced autonomous activity (after EGTA addition). n=3 replicates. % of total activity (no EGTA addition). **(E)** Western Blot - Purified CaMKII Cys273-Cys290 and Cys273-Ala287-Cys290 were incubated under the conditions indicated in the figure and probed with anti-CaMKII antibody or anti-Thr287 phosphorylated antibody (representative image of n=3 replicates).

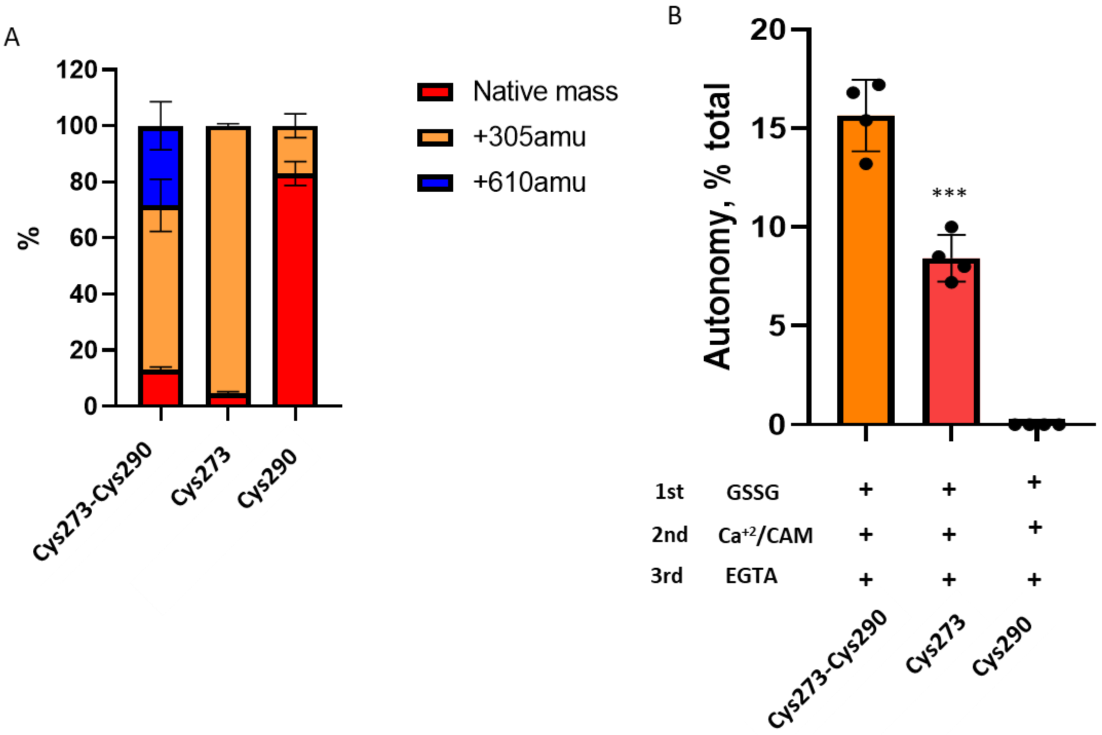

Supplementary figure 5: Glutathionylation of CaMKII Cys273-Cys290 and Cys273 induces autonomy. Purified CaMKII Cys273-Cys290, Cys273, and Cys290 were incubated with oxidized glutathione (GSSG). **(A)** The mass of the proteins before and after the incubation with GSSG was measured using HPLC-MS/MS. Each glutathione attached to the protein adds 305amu. **(B)** CaMKII mutants after incubation with GSSG autonomous activity (after EGTA addition). n=4 replicates. % of total activity (no EGTA addition). *** P=0.005 compared with Cys273-Cys290 mutant by unpaired t-test.

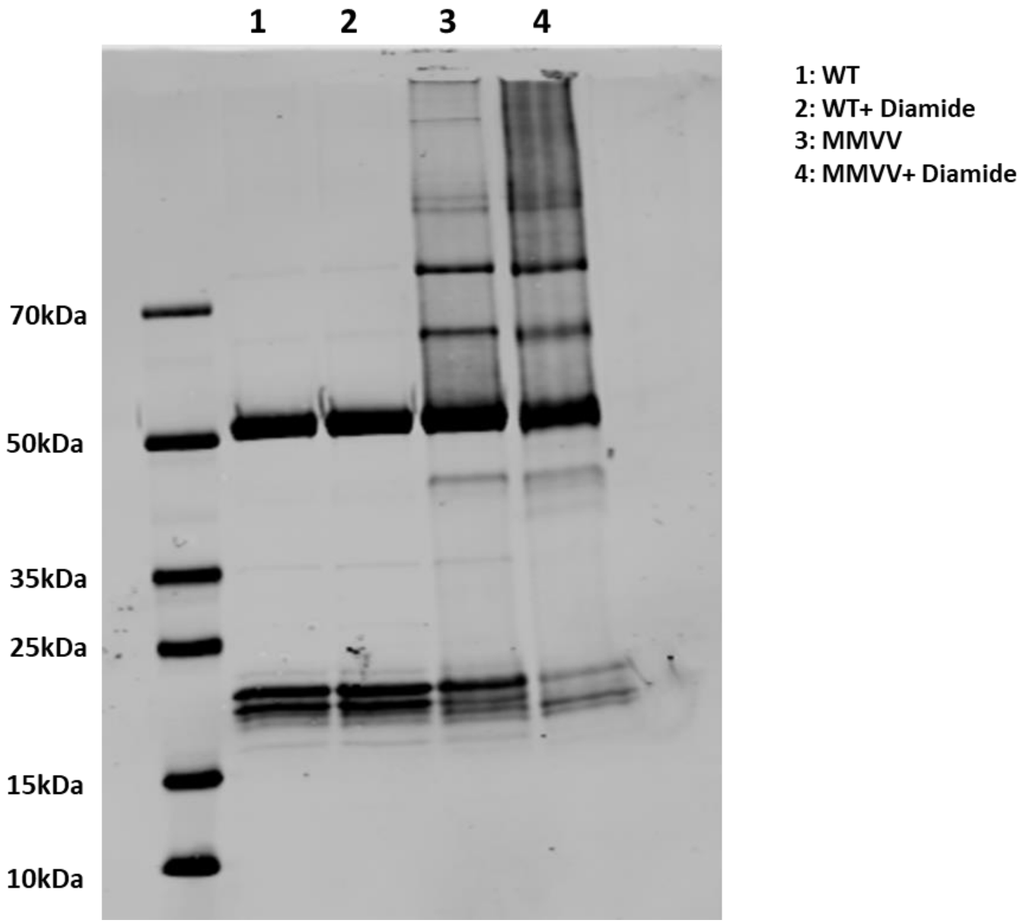

Supplementary figure 6: Purified MMVV CaMKII shows aggregation and degradation. Purified CaMKII WT and MMVV mutant were incubated or not with diamide and probed with anti-CaMKII antibody. Representative image of n=3 replicates.

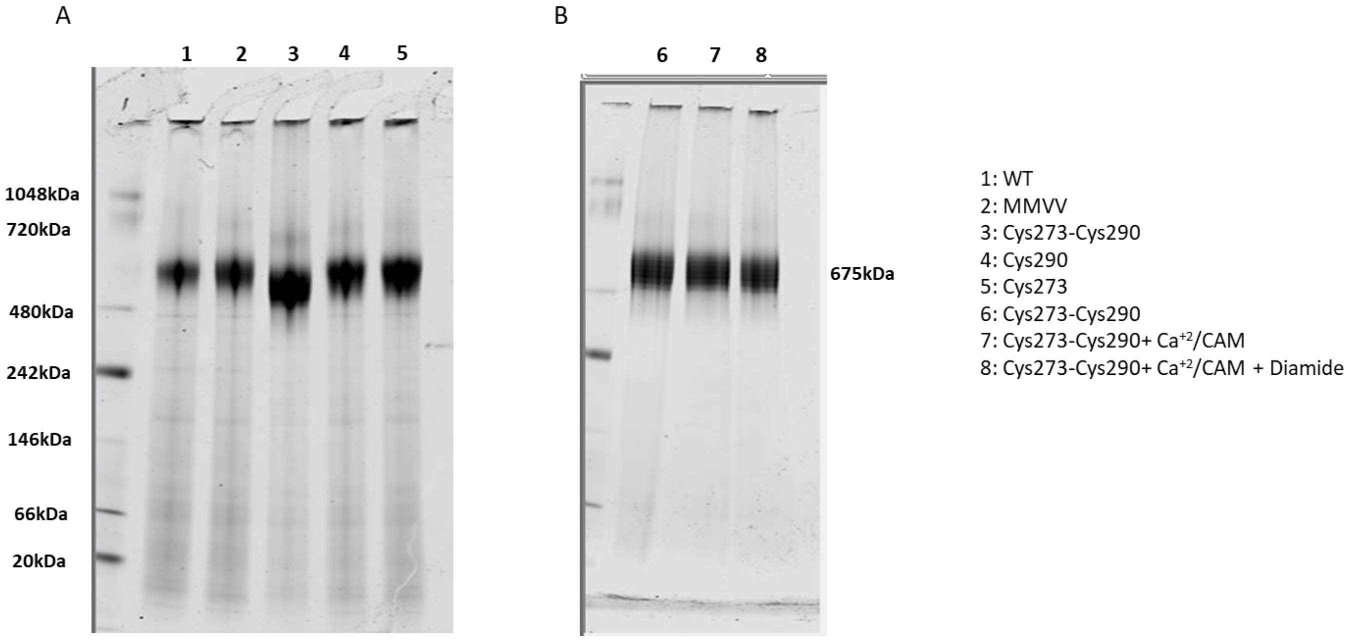

Supplementary figure 7: CaMKII dodecamer formation. (A) Blue Native gel showing CaMKII dodecamer formation by all CaMKII mutants used in this work and WT. **(B)** Cys273-Cys290 dodecamer after incubation with Ca^{+2}/CAM or Ca^{+2}/CAM + diamide.

A

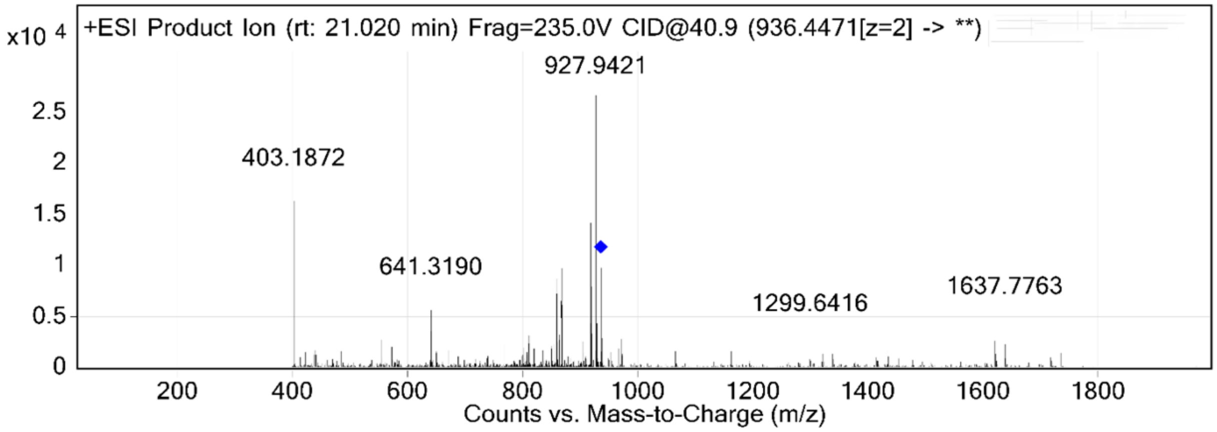

B

Cys273-→Cys290 $H_1P_2W_3I_4C_5Q_6R_7$-→$Q_8E_9T_{10}V_{11}D_{12}C_{13}L_{14}K_{15}$			
y'	Residue	Calculated m/z	Observed m/z
14	Pro	1734.83	1734.84
13	Trp	1637.78	1637.78
12	Ile	1451.70	1451.70
11	Cys	1338.61	1338.61
Cys290-→Cys273 $Q_1E_2T_3V_4D_5C_6L_7K_8$-→$H_9P_{10}W_{11}I_{12}C_{13}Q_{14}R_{15}$			
y'	Residue	Calculated m/z	Observed m/z
14	Glu	1743.83	1743.82
13	Thr	1614.79	1614.77
12	Val	1513.74	1513.74
11	Asp	1414.67	1414.67
10	Cys	1299.64	1299.64
9	Leu	1197.64	1197.60
8	Lys	1084.56	1084.50

Supplementary Figure 8: MS/MS peak assignments for the Cys273-Cys290 disulfide-linked dipeptide. (A) The MS/MS spectra of disulfide-linked peptides is very complex because both peptides contribute ions, a, b, x, y', z' and others. Analysis can be simplified by first calculating the y' m/z for both peptides and then identifying the peaks observed in the MS/MS spectrum. **(B)** Expected peaks were calculated by GPMAW from the carboxyl terminus to the amino terminus. As shown in the table, the dipeptide was unambiguously identified.

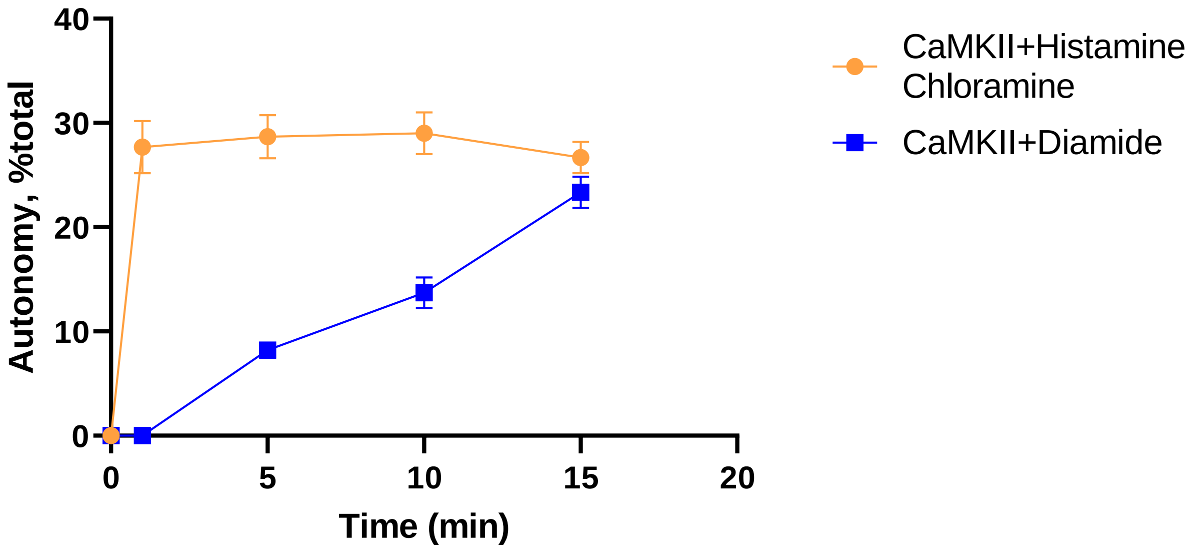

Supplementary figure 9: Time course of CaMKII autonomy induced by diamide or histamine chloramine. Purified CaMKII Cys273-Cys290 was incubated with diamide (blue) or histamine chloramine (yellow) for 1, 5, 10 and 15 min and autonomous activity (after EGTA addition) was measured. n=3 replicates. % of total activity (no EGTA addition).

Cys 273 to Cys	Mass	Z	m/z	Present?
31	1,316.59	2	659.30	No
65	1,362.64	2	664.33	No
116 & 127	4,400.03	6	734.35	No
200	4,828.32	6	805.73	No
273	1,872.88	2	937.45	No
290	1,870.88	2	936.45	Yes
373	3,666.72	4	917.69	No
428	4,016.08	5	804.22	No

Supplementary table 1: The disulfide links two tryptic peptides, residues 268-274 with Cys273 and residues 284-291 with Cys290. Mass of Cys273 linked with all the other cysteines containing tryptic peptides. The listed m/z was used to obtain the EIC in figure 4A.
